# Effect of Shade on Agro-Morphological Parameters and Weed Flora of Saffron (*Crocus sativus* L.) Cultivation in the Semiarid Zone of Eastern Morocco

**DOI:** 10.1155/2022/9954404

**Published:** 2022-02-15

**Authors:** Ibtissam Mzabri, Maria Rimani, Khadija Charif, Noureddine Kouddane, Abdelbasset Berrichi

**Affiliations:** ^1^Laboratory for Agricultural Production Improvement, Biotechnology and Environment, Faculty of Science, Mohammed First University, Oujda, Morocco; ^2^Laboratory Bioresources, Biotechnology, Ethnopharmacology and Health, Faculty of Sciences, University of Mohammed First, Oujda, Morocco

## Abstract

Saffron (*Crocus sativus*) has been an important medicinal plant since ancient times. This study aimed to seek the optimal light intensity for saffron growth by quantifying the effects of different shade levels on yield, vegetative growth, and weed development in the eastern region of Morocco. The plants were grown for 24 months in full sun (control) and 30%, 50%, and 70% shade. Overall, the results showed that shade positively affected the yield and vegetative growth parameters of saffron plants, with the highest yield (0.61 g/m^2^) and number of leaves (105 leaves/tuft) recorded when the plants were exposed to light shade (30%). The color of the leaves under the 70% shade levels was dark green. The results from the underground part showed that shade is positively correlated with the weight and diameter of daughter corms where the 70% shade recorded the highest values of weight (65 g) and percentage of large diameter corms (39%). As for weed density, this parameter was significantly affected by shade. The lowest weed density was recorded for the 70% shade treatment. In conclusion, 30% shade is suggested as optimal light irradiation for saffron cultivation.

## 1. Introduction

*Crocus sativus* L. is an autumnal flowering geophytic plant of the family Iridaceae [1]. This plant is cultivated in many countries such as Iran, Greece, Spain, Turkey, and Morocco [2]. Its well-known product is called saffron, which is considered among the most valuable and irreplaceable spices worldwide [3]. Saffron is highly coveted for its beauty, aroma, and medicinal properties [4]. In 2018, the saffron plantation in Morocco was carried out on an area of about 1,880 ha with a production of nearly 6.4 t, making Morocco the fourth largest saffron producer in the world [5]. The adaptation to various ecosystems [6] and the economic importance [7] of saffron cultivation in Morocco call for special attention to improve production, export, and merchandising techniques.

As saffron is a crop of high economic value, much in-depth research has been carried out worldwide to increase yield under difficult growing conditions in some countries [8]. Fluctuating biotic and abiotic factors are a major challenge of climate change for good quality agricultural production [9]. Climate change is also considered to be the main cause of the distribution of weeds in different cropping systems, leading to a complex crop-weed interaction [10]. Despite the technological progress made in improving farming practices (irrigation, fertilization, plant protection measures), the climate remains one of the key factors in agricultural productivity. Solar radiation is one of the main abiotic factors for agricultural production [11]. The study of microclimate modification using different shade nets revealed that crops behaved differently under shaded conditions [12]. The plants exposed to low irradiation generally exhibit shade avoidance strategies and/or tolerance mechanisms, such as shoot elongation, increased leaf area with low leaf mass per unit area, and changes in chlorophyll a/chlorophyll b ratios [13]. In general, the acclimatization of plants to different light intensities depends on the genotype and environmental conditions of the plant. As a result, there is a lack of information dealing with the shadow effect on the production of saffron in the world. Based on these facts, this study was carried out to evaluate the effect of shade on the agro-morphological parameters of saffron on the one hand and on the distribution and density of weeds in this crop on the other hand. Overall, the main objective of this study was to provide agronomists with information to develop appropriate planting schemes in which saffron plants receive an optimal light intensity for their growth and to reveal the negative effect of shade on weed development, especially since weeding is considered to be an important and costly part of the overall saffron cultivation practices.

## 2. Materials and Methods

### 2.1. Site Characteristics

**Figure 1:**
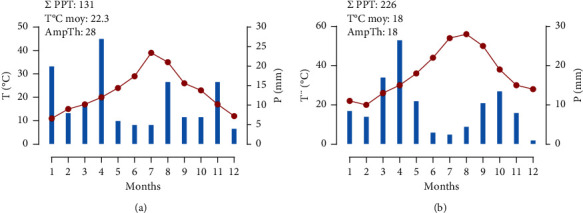
Monthly weather data from the experimental station of the Faculty of Science in Oujda, for the experimental period January-December 2016 (a) and January-December 2017 (b). Σ PPT: sum of precipitation, AmpTh: temperature range, *T*°C moy: mean temperature.

The experiment was conducted in the open field at the experimental research station of the Faculty of Science of Oujda, located at an altitude of 661 m and 34° 39′06–71″ north and 01° 53′58–80″ west (GPS BackTrack Bushnell). The soil has a well-drained silty clay texture. The semiarid climate is characterized by temperate winter. The precipitation was modest for most of the trial period, especially during the first year of the trial (131 mm). The saffron requirements were supplemented by drip irrigation during dry periods. The minimum temperatures were recorded during January (−0.4°C and 1°C), respectively, for the years 2016–2017 (Figure 1).

### 2.2. Plant Material and Growing Conditions

The saffron corms, 2.5 cm in diameter, used in this trial come from a saffron plantation in the experimental research station of the Faculty of Science of Oujda planted on September 21, 2015. The plantation was carried out in August 2016 in the open field under the green plastic shade with three levels of shade (30%, 50%, and 70%).

The shade cloth was fixed to metal arches forming mini-tunnels of dimensions (width 1 m × height 0.4 m × length 3 m). The level of shading was measured using a luxmeter (Lutron; LM-8000). The growth and production of saffron under these shading levels were compared to those planted in full sun (without shade nets).

### 2.3. The Measured Parameters

Growth parameters, yield components, weed density, and dry matter were recorded throughout the crop cycle. Five plants/treatments were randomly selected and used to count the number of leaves and measure leaf height. The color evolution of the leaves of the different treatments was monitored monthly by measuring the color scale *L*∗, *a*∗, and *b*∗ using a MiniScan XE™ Spectrophotometer (Hunterlab Inc., Reston, VA, USA) equipped with a D65 (daylight) lamp as the light source (wavelength between 400 and 700 nm). The color space is based on a Cartesian representation with three orthogonal axes: *L*∗, *a*∗, and *b*∗ [14, 15]. L represents lightness (*L* = 0, black, and *L* = 100, colorless); *a*∗, a color component: green/red (*a*∗ > 0, red, and a∗ < 0, green); and *b*∗, a color component: blue/yellow (*b*∗ > 0, yellow, and *b*∗ < 0, blue). At the end of the test, the aerial dry biomass, expressed in grams, is obtained by weighing the dry matter after steaming at 80°C for 48 h of the previously weighed fresh matter.

For the stigma yield, the flowers of each treatment were harvested very early in the morning after the pruning operation, which consists of cutting through the nails the lower part of the flower just below the point of attachment of the stigma. The stigmas were then measured and spread out on flat containers in the shade for a few days for drying, after which the dry weight was obtained by weighing the dry stigmas [16].

The characterization of the weed flora associated with this crop under shaded conditions was carried out using the “round-field” sampling method, which was considered the most appropriate method for this context and consists of going through the plot in different directions until the discovery of a new species [17]. This method has the advantage of taking into account the heterogeneity of the plot and thus makes it possible to take into account rarely, rapidly expanding species or species indicative of certain characteristics of the environment.

The degree of weed infestation (weed density and dry weight of weeds) was assessed by counting and weighing (dry weight of weeds) in a quadrat of 1 m × 1 m per plot. The nomenclature adopted is that of the flora of North Africa [18].

### 2.4. Experimental Design and Statistical Analyses

The experimental design adopted is a complete randomized block (BAC), it consists of 3 blocks with a total of 120 tufts of saffron (10 tufts/treatment × 4 treatments × 3 repetitions), the blocks indicate the repetitions, and the sub-blocks represent the treatments. The statistical analysis of data was carried out using the analysis of variance (ANOVA) procedure on “GraphPad Prism for Windows version 7.” The averages were compared with Duncan's test at 5% level.

## 3. Results

### 3.1. Saffron Yield

Yield is considered to be the result of the coordination of yield components such as the number of flowers, weight, and length of stigmas. The results of yield components are shown in Table 1. The analysis of variance indicates that the number of flowers, stigma yield, and stigma length was significantly affected by shade throughout the experiment. Comparison of the means showed that the treatments with the highest sun exposure (control and 30%) had the highest number of flowers with respective means of 1.4 and 2 flowers/corms in the first year of the experiment and 2 and 2.3 flowers/corms in the second year. On the other hand, the length of the stigmas increased considerably for the 70% shade treatment compared with those grown in full, light, and mid-sun. The stigmas/m^2^ yield showed that light shade (30%) resulted in the highest yield with 0.61 g/m^2^, an increase of 5% compared with the control. The difference observed between the treatments in terms of earliness and harvest time was not statistically significant (*p* < 0.05).

### 3.2. Growth and Morphology of Plant

**Figure 2:**
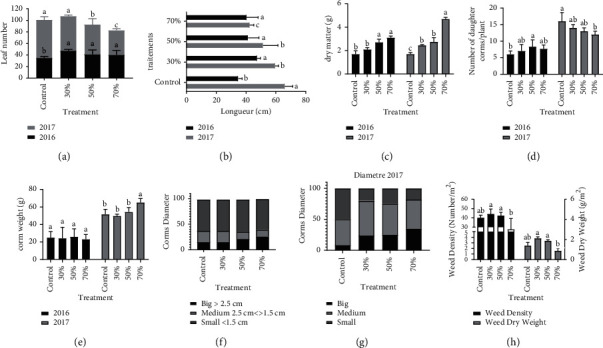
Effects of shading on the morphological parameters of the rudder. (a) Number of leaves; (b) leaf area; (c) dry matter; (d) number of thread corms; (e) weight of thread corms; (f) and (g) diameter of wire corms; and (h) density and dry weight of weeds associated with saffron cultivation. The data are the average of the five measurements made during the experiment. Different letters indicate significant differences between treatments (*p* < 0.05).

Leaf morphology was significantly affected by shade treatments. The number of leaves changes from year to year, but in general, the number of leaves/plants in the shade of 30% was significantly higher than that of the other treatments (*p* < 0.05) throughout the trial period (Figure 2(a)). The plants that were grown under 50% and 70% shade had the largest leaf area, while the leaves of plants grown in full sun were the smallest (Figure 2(b)). The same finding was made for the dry matter of the aerial part (Figure 2(c)) with the highest values being found in the case of the 70% shade treatment (*p* ˂ 0.001) followed by the 50% and 30% shade treatment, which showed increases of 63%, 38%, and 31%, respectively, compared with the control treatment in the second year of the trial. The effect of shade on saffron leaf color is shown in Table 2. The results showed that the rate of shading significantly influenced the color of the leaves (*p* < 0.05). The *L*∗ values of the leaves decreased with increasing shading rate, which explains why the color of the leaves became darker when planted under high shading rates. Similarly, the *a*∗ values showed a negative correlation with the shading rate of −07.86, −10.82, −09.99, and −09.51, respectively, for the control, 30%, 50%, and 70% treatments. The green color of the leaves tends to decrease in the plants most exposed to the sun. The increase in the shading rate does not seem to be too much affected by the *b*∗ values, which vary between 8.45 (control) and 12.68 (30%). Overall, the leaves of plants grown under artificial shade were darker with intense green color, while the leaves of those grown in full sun were lighter with a less intense green color.

### 3.3. Underground Part Parameters

Saffron production is directly influenced by the size of the corms at the time of planting. According to the results, the number (Figure 2(d)), weight (Figure 2(e)), and diameter (Figures 2(f) and 2(g)) of daughter corms were also strongly affected by shade. The number of corms produced increased in the second year of the experiment for all treatments. In addition, the maximum number of daughter corms was obtained in the case of plants exposed entirely to the sun with a number that approached 15 corms/plants in the second year.

The increase in the number of daughter corms in the second year of the experiment increased the weight of the corms for all treatments, with the greatest increase recorded for the most shaded treatment (70%). However, the comparison of the means did not show a significant difference between the 50% and 30% treatments, and the control (*p* > 0.05).

The results show that the percentage of the different diameter categories varies according to the treatment applied. The plants growing under 70% shade showed a dominance of the “large (*Ø* > 2.5 cm) and medium diameter” categories (1.5 < *Ø* < 2.5 cm), especially in the second year of the experiment, while the control plants showed a dominance of the “small diameter” category (*Ø* < 1.5 mm), which exceeded half in the second year.

### 3.4. The Nature, Density, and Dry Weight of Weeds

**Figure 3:**
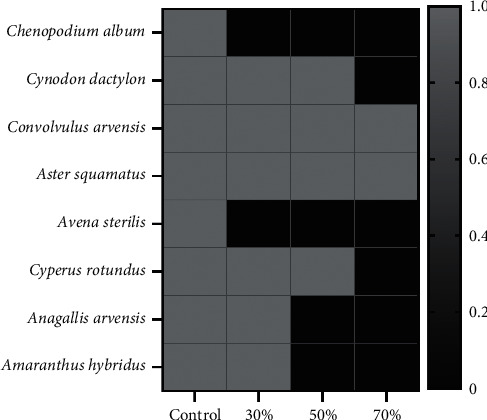
Weeds present in the different shaded areas (control, 30%, 50%, and 70%).

The nature, density, and dry weight of the weeds were significantly affected by the variation in light intensity. Some weed species are indifferent to the variation of light intensity such as *Convolvulus arvensis, Cyperus rotundus, Aster squamatus,* and *Conyza bonariensis,* which grew in a normal way in the four treatments studied. However, *Chenopodium album, Anagallis arvensis,* and *Avena sterilis* were found only in the treatments exposed to sunlight (control/30% shade) (Figure 3). Weed density and dry weight showed a difference between treatments (Figure 2(h)). The lowest values were recorded in the case of the treatment, which received only 30% light (70% shade) with a density of 28 plants/m^2^ and a dry weight of 1.5 g/m^2^ and with a dominance of *Aster squamatus*. While the treatments that receive 30% and 50% shade showed comparatively higher weed density and dry weight.

### 3.5. Effect on the Vegetation Cycle

The annual cycle of saffron has five stages: emergence, flowering, leaf development, bulb development, and dormancy. The time and duration of each stage depend on the climatic conditions. It is therefore in autumn when all other plants fall asleep to escape the harsh weather conditions that saffron flowers. It goes into dormancy in spring, and its foliage disappears completely. The field observations show that shade treatment affects the rate of dormancy. The first signs of leaf yellowing were observed as early as the last decade of March in the control treatment, followed by the 30% treatment and finally the 70% shade treatment, which externalized the signs of dormancy from the first week of April.

## 4. Discussion

The yield increases in partially shaded treatment could be related to changes in shaded plot conditions, especially since colored shade nets can alter the light spectrum [19] and increase light scattering [20]. Also, partial shading has globally improved the water status of the plants by decreasing temperature and consequently evapotranspiration (data not shown). For this study, 30% shade also increased vegetative growth, especially the number of daughter corms, which could contribute to the increase in the number of flowers. These findings for saffron are comparable to those from the bell pepper crop [21] and chili pepper [22] in which the yield parameters were increased by the partially shaded conditions [23]. The results also revealed a significant reduction in yield at a shading level of 70%, which in perfect agreement with a soybean study showed that seed yield decreased significantly under severe shading conditions compared with normal conditions. This decrease could be explained by the fact that the severe shading conditions (70%) favored the vegetative growth parameters, especially the aboveground and belowground biomass. The hypothesis of plant-biomass sharing under shaded conditions demonstrated a preference of translocation of photosynthesis for the formation of the vegetative part of the saffron plant to the detriment of floral initiation and flower production.

The results showed that the length of the stigmas increased considerably for the 70% shade treatment compared with those grown in full sun. This reaction suggests that floral allometry (changes in floral parameters in relation to overall flower size) may vary from one light environment to another. Weinig [24] reported that the petal length of *Arabidopsis thaliana* increases under experimental shade conditions. Similarly, Kingsolver et al. [25] found that under shaded conditions there is an increase in the length of the stamens and a reduction in the length of the pistil.

The severe shade (50% and 70%) embroidered a low stigma yield, and this suggests that a partial shade rate (30%) could be recommended to optimize the management of the saffron crop. These results were associated with a low yield of stigmas, and this suggests that a partial shade rate (30%) could be recommended to optimize the management of the saffron crop.

Light rays are considered to be one of the most important abiotic factors for plant production [26]. In horticulture, the ultimate goal of using shade nets is to modify the quality and quantity of the light spectrum. This change can act as a physiological tool to modify crop microclimate and improve plant growth and productivity [27]. The creation of a microclimate using different levels of shading has resulted in a remarkable agro-morphological response of saffron plants. In general, shaded plants undergo changes to maximize light uptake and transduction.

It showed a slight increase in leaf count during the first year, while in the second year the number of leaves per plant was higher for 30% shade and then decreased with increasing shade levels. Similar results were observed on *Capsicum annum* [21] and *Salvia officinalis* [28], for which the number of leaves tended to decrease with increasing shade intensity.

The results obtained revealed a significantly higher leaf area under 70% shading, followed by 50% and 30%, for both years of the trial. Our results are consistent with the study showing that *Salvia officinalis* grown under a 50% shade level showed maximum leaf area [27]. Similar results were found on shaded tomato and bell pepper plants, which expressed longer internodes and larger leaves [27, 29]. Leaf area is an important factor in mechanisms such as radiation interception and water and energy exchange [30]. A large leaf area has led to increased chlorophyll levels in shade-grown saffron plants (unpublished data), which could increase photosynthesis as reported on ginger cultivation [31].

Shading also resulted in a significant increase in the biomass of the aerial part per plant (*p* < 0.05), and the highest values were obtained under 70% shade. These light levels favored photosynthesis, which led to an increase in biomass production [32] as the increase in the rate of photosynthesis is one of the main factors in the production of plant biomass [33]. These results are in agreement with De Carvalho Gonçalves et al. [34] who reported that conditions of intermediate lighting (about 50% of full sunlight) have led to higher levels of biomass production in some species. Similarly, Pegoraro et al. [35] found that dry mass was highest at 30% shade. However, the plants of several species grown under low-light conditions were less productive than those grown under high-light conditions [27, 35].

According to the literature, the plants in the shade show changes in leaf anatomy, color, and morphology [36]. Light and temperature are the two climatic variables that strongly affect leaf color [37]. The use of spectrophotometric or colorimetric techniques is in full expansion because they require less time and provide easily comparable results [14]. The CIELAB method uniformly covers the entire visible spectrum of the human eye [15, 38]. In general, the determination of color quality is based on luminosity (*L*∗), greenish to reddish color (*a*∗), and blue to yellowish color (*b*∗). Leaf color can be used to identify stress caused by adaptation to environmental change [39]. Saffron plants growing in the shade had dark green leaves, while those grown in full sun showed leaves with a light green coloration. These results are consistent with those of Ilić et al. [40], which showed that lettuce leaves grown in shade had a more intense green tone compared with lettuce grown in the open field [40], while sage plants grown under 50% and 70% shade showed lighter leaves compared with those grown in full sun [27]. The green color change (*a*∗ values) was probably related to chlorophyll degradation. The decrease in yellowing (*b*∗ values) of the leaves of saffron grown in full sun was probably due to the degradation of the carotenoid compound [41].

In general, the reaction of saffron plants to the shade resulted in a change in growth and development to receive a sufficient amount of light. The same results were found in fragrant geranium (*Pelargonium graveolens*), sage (*Salvia officinalis*), and bottle gourd (*Lagenaria vulgaris*) in which the highest growth parameters were found under shady conditions [27, 42, 43].

Under low-light conditions (70% shade), a decrease in the number of corm threads was observed, especially during the second year of the trial. Similar results were found by Wurr et al. [44] on potato where the 70% shade level resulted in a decrease in tuber numbers. This decrease could be due to the preferential use of assimilates for leaf mass rather than the number of corm threads to provide an adequate level of photosynthesis. The decrease in the number of corms in shaded plants was compensated by a higher total weight of the underground part and a preponderant fraction of large and medium diameter corms. Similar trends were also reported on ginger [45–47] and on turmeric [48]. This could be due on the one hand to a higher photosynthetic activity stimulated by significant vegetative growth (leaf area, aboveground biomass) under favorable soil temperature and higher relative humidity and on the other hand to a decrease in the rate of photooxidation and efficient translocation of photosynthates in shaded conditions [49].

It should be noted that severe artificial shading (70%) has caused the suppression of certain weeds (e.g., *Anagallis arvensis* and *Avena sterilis*). The same observation was made by [50], on the suppression of *Tradescantia fluminensis* (Commelinaceae) under shaded conditions. In addition, for most annual plants, 90% shade reduced seed production by up to 90%. Shade reduced the production of purple nutsedge tubers by 89%. However, some species persisted at a severe shade rate (*Convolvulus arvensis, Cyperus rotundus, Aster squamatus*, and *Conyza bonariensis*). It should be noted that *Convolvulus arvensis* L. grows in a wide range of conditions from full sun to full shade [51]. The leaf area of *Cyperus rotundus* grown under 50% and 70% shade was 21.45 and 46.05% higher, respectively, than that of plants grown in full sun [52]. The results proved that some weeds are not only adapted to intense light, but also are more capable of adapting to extreme light variations, especially at high shade rates, by exhibiting plastic responses that minimize growth-limiting effects [53]. In the same context, Godara et al. [54] found that in 70% and 90% shade, the height and the leaf area of Texas weed were increased by 28% and 20%, respectively, suggesting that the weed appeared to counteract the adverse effect of a shade. In general, studies have shown that dicotyledons are less sensitive to shade than monocotyledons, which are photosynthetically less flexible in shaded environments [46]. On the other hand, partial shading resulted in higher weed density and dry weight, a result affirmed by several studies on many weed species, which concluded that partial shading increases chlorophyll content and leaf height and area [55, 56]. In line with the results found, it is suggested that 70% of shading can be considered a limiting factor in weed density in saffron cultivation. Nevertheless, weed control depends not only on the shade tolerance of the target weed but also on the relative tolerance of the coexisting vegetation.

The results found in this study showed that shade slows down the speed at which saffron plants go into dormancy. The observed effect could be related to changes in the conditions of the shaded plots, especially the temperature, which is generally cooler than that in the full sun. Our observations are consistent with those of Molina et al. [57] who reported that in Spain, saffron entered dormancy at different times depending on the average air temperature. The leaves dried earlier in places where the average temperature was higher than that in sites with cooler temperatures.

## 5. Conclusion

The studies on the impact of different shade treatments on saffron plants are almost nonexistent. This experiment aimed to determine the threshold level of shade for better growth and development of saffron under the conditions of the semiarid region of Morocco to propose saffron as an intercrop. The results showed that saffron needs a partial shade of 30% to reach its maximum growth and especially the yield in stigmas, and the latter parameter is considered the most important for saffron cultivation. The results also showed that saffron can regulate its metabolism and adapt to more severe levels of shading. The increase in the shade (50% and above all 70%) significantly improved morphological parameters and particularly leaf area, aerial biomass, weight, and diameter of daughter corms, something that may capture the attention of saffron seed producers. This experiment also confirmed the favorable advantages of shade on the decrease in the weed population. In addition, it could be recommended to the farmer to adopt a planting density that provides sufficient shade to inhibit the germination and development of certain weeds and thus ensure better management of water resources, especially in dry areas where saffron is grown. Overall, these results imply that farmers should ensure a partial shade rate of 30% to maximize saffron production. Similarly, this plant can be programmed into cross-cultural planting schemes. In the eastern region of Morocco, saffron could be intercropped in fruit orchards with deciduous foliage (rosaceous) or low density that allows saffron to receive sufficient light.

## Figures and Tables

**Table 1:** Effect of different shade treatments (control, 30%, 50%, and 70%) on the harvest period, number of flowers, weight, and length of stigmas. The values are the averages of 3 replicates.

Shade treatments	Flowering duration (day)		Number of flowers/corms		Saffron stigma yield (g/1 m^2^)		Stigma length (cm)	
	2016	2017	2016	2017	2016	2017	2016	2017
Control	11a	13a	1.4ab	2 a	0.32bc	0.58b	3 b	3.4b
30%	13a	12a	2 a	2.3a	0.45a	0.61a	3.2b	3.3b
50%	10a	10a	0.8b	1.1b	0.29c	0.55c	3.2b	3.5b
70%	10a	09a	0.9b	1 b	0.32bc	0.52c	4.3a	4.6a

Means with the same letter within a given treatment are not significantly different at *p* = 0.05.

**Table 2:** Color coordinates of saffron leaves grown in full sun (0%) and 30%, 50%, and 70% shade.

Treatments	*L*∗	*a*∗	*b*∗
Control	52,77c	−07.36a	08,45a
30%	44,46b	−10.60c	12,62b
50%	40,70b	−09.99b	11,33b
70%	42,21a	−09.51b	10,96b

Means with the same letter within a given treatment are not significantly different at *p* = 0.05.

## Data Availability

The data used to support the findings of this study are available from the corresponding author upon request.
